# Barriers and facilitators to recruitment of underrepresented research participants: Perspectives of clinical research coordinators

**DOI:** 10.1017/cts.2023.611

**Published:** 2023-08-18

**Authors:** Marie E. Heffernan, Leo Barrera, Zecilly R. Guzman, Emily Golbeck, Aneta M. Jedraszko, P. Toddie Hays, Keith A. Herzog, Richard T. D’Aquila, Michael G. Ison, Susanna A. McColley

**Affiliations:** 1 Department of Pediatrics, Ann & Robert H. Lurie Children’s Hospital of Chicago, Northwestern University Feinberg School of Medicine, Chicago, IL, USA; 2 Mary Ann & J. Milburn Smith Child Health Outcomes, Research, and Evaluation Center, Stanley Manne Children’s Research Institute, Ann & Robert H. Lurie Children’s Hospital of Chicago, Chicago, IL, USA; 3 Department of Pediatrics (Neonatology), Ann & Robert H. Lurie Children’s Hospital of Chicago, Chicago, IL, USA; 4 Clinical and Translational Sciences Institute (NUCATS), Northwestern University, Chicago, IL, USA; 5 Department of Medicine (Infection Diseases), Northwestern University Feinberg School of Medicine, Chicago, IL, USA; 6 Respiratory Diseases Branch, National Institute of Allergy and Infectious Diseases, Rockville, MD, USA; 7 Department of Pediatrics (Pulmonary and Sleep Medicine), Ann & Robert H. Lurie Children’s Hospital of Chicago, Northwestern University Feinberg School of Medicine, Chicago, IL, USA

**Keywords:** Research recruitment, research participation, special populations, underrepresented groups, clinical research coordinators, research equity, clinical trials

## Abstract

**Background:**

Insufficient recruitment of groups underrepresented in medical research threatens the generalizability of research findings and compounds inequity in research and medicine. In the present study, we examined barriers and facilitators to recruitment of underrepresented research participants from the perspective of clinical research coordinators (CRCs).

**Methods:**

CRCs from one adult and one pediatric academic medical centers completed an online survey in April-May 2022. Survey topics included: participant language and translations, cultural competency training, incentives for research participation, study location, and participant research literacy. CRCs also reported their success in recruiting individuals from various backgrounds and completed an implicit bias measure.

**Results:**

Surveys were completed by 220 CRCs. CRCs indicated that recruitment is improved by having translated study materials, providing incentives to compensate participants, and reducing the number of in-person study visits. Most CRCs had completed some form of cultural competency training, but most also felt that the training either had no effect or made them feel less confident in approaching prospective participants from backgrounds different than their own. In general, CRCs reported having greater success in recruiting prospective participants from groups that are not underrepresented in research. Results of the implicit bias measure did not indicate that bias was associated with intentions to approach a prospective participant.

**Conclusions:**

CRCs identified several strategies to improve recruitment of underrepresented research participants, and CRC insights aligned with insights from research participants in previous work. Further research is needed to understand the impact of cultural competency training on recruitment of underrepresented research participants.

## Introduction

Recruitment is a significant challenge in the conduct of clinical research studies. A review of ClinicalTrial.gov registered studies found that low recruitment was the primary reason for study closure prior to completion [1–4]. Other studies required extensions to meet their target recruitment goal, resulting in additional costs and delays in bringing treatments to market [3]. Delayed recruitment poses a serious problem to the success of clinical research studies and the discovery of new therapeutics and interventions.

In addition to overarching challenges with recruitment, recruitment of groups underrepresented in medical research (henceforth “underrepresented groups”) is a challenge that threatens the generalizability of research findings and compounds inequity in research and medicine [5,6]. Historically, Black/African American and Latinx/Hispanic populations have been underrepresented in clinical research studies. Despite some improvements [7], the pattern continues [8–13]. In a 2017 review of enrollment in all therapeutic cancer trials between 2003 and 2016, the proportion of White participants was 83%, whereas only 66% of the US population was White in the 2010 US Census [8]. Additionally, an FDA report of participant demographics in clinical trials for drugs and biologics approved in 2020 indicated that 75% of participants were White, but only 62% of the US population was White in the 2020 US Census [9]. A recent report from the National Academies of Science, Engineering and Medicine notes that although there has been progress in recruiting White women to research studies, the last three decades have seen little progress in increasing research participation among racial and ethnic minority population groups [6]. Insufficient recruitment of underrepresented populations occurs in both interventional and observational research [14]. Initiatives and policies have been enacted to improve representation among women and racial and ethnic minorities in clinical research (e.g., 21^st^ Century Cures Act; NIA Office of Special Populations; American Thoracic Society) [15–17].

Efforts to improve clinical research recruitment have included research on the barriers and facilitators to research participation. A review on this topic identified themes, such as prospective participants’ attitudes toward research and the healthcare system, logistic obstacles to participation, and characteristics of the study [18].

Other research has explored differences in barriers by participant characteristics. For instance, Non-Hispanic Black participants were more likely to report the role of trust/mistrust in their decision to participant in clinical research studies, while Hispanic participants reported that incentives play a more key role in their decisions [19,20]. A recent review of barriers to representation in research noted that among racial and ethnic minority groups, salient barriers included trust and confidentiality, lack of access to available studies, and challenges with participant contact and scheduling [21]. Other key drivers of underrepresentation in research include language barriers such as unavailability of translated study materials, competing work and caregiving responsibilities, and costs of participation such as transportation [17,22,23].

Another potential cause for low recruitment of underrepresented groups is implicit bias. Implicit biases are attitudes towards people or groups of people that are automatic or unconscious [24]. Previous research has found that research staff, including physicians and principal investigators, demonstrated bias toward and stereotyping of minority participants [25]. This could result in lower recruitment rates among underrepresented populations.

The extant literature on challenges to clinical research study recruitment and inequities in clinical research have primarily examined these issues from the perspectives of prospective participants, with relatively fewer studies having examined these issues from the perspectives of clinical research staff involved in recruitment (henceforth “clinical research coordinator” or “CRC”). Studies that have explored the CRC perspective have either been largely qualitative in nature or have focused on a specific disease area [26–28]. One exception was a large survey study of research professionals from a variety of research roles (e.g., investigator, CRC, and research nurse). Results of this study indicated that a language barrier was the strongest barrier for minority recruitment; however, results were not compared across research roles [22]. We focused on CRCs because they are frontline staff in clinical research recruitment. Therefore, they represent one of the early steps in the research process that can be targeted to improve recruitment both generally and recruitment of underrepresented populations specifically.

The aims of the current study were to gather perspectives from CRCs involved in clinical research study recruitment across multiple study types to understand their views on barriers and facilitators to recruitment, especially when recruiting study participants from underrepresented groups, and to explore implicit bias as a potential factor about which CRCs may have less conscious awareness.

## Materials and Methods

The present research was conducted at two academic medical centers located in Chicago, Illinois, and affiliated with Northwestern University (NU) Feinberg School of Medicine and the Northwestern University Clinical and Translational Sciences Institute (NUCATS). The first, Northwestern Memorial Health (NMH), is an adult-serving academic medical center. The second, Ann & Robert H. Lurie Children’s Hospital of Chicago (LCH), is a pediatric medical center serving children, adolescents, and young adults from birth to age 25 years.

### Recruitment

Survey invitations with a link and QR code were sent out via work-based email listservs from May 17–27, 2022, to employees of NU, NMH, and LCH who were identified as being part of the clinical research workforce based on their membership in clinical research listservs maintained by NUCATS and the LCH Research Development Office. After the initial invitation, two reminders were sent via listservs and posted in a digital chat-based workspace.

### Survey Instrument

Topics for the survey were generated by a multi-institutional and interdepartmental panel of CRCs, researchers, and research administrators. Survey items were developed and refined by a multidisciplinary team, including experts in survey development, clinical research operations, and diversity, equity, and inclusion. We assessed CRCs’ perceptions of the following factors that may impact clinical research study recruitment: language and translations (e.g., having translated study documents), employee training (e.g., attending a cultural competency training), participant incentives (e.g., offering gift card incentives), study location (e.g., reducing required in-person visits), and participant research literacy (e.g., availability of educational videos about research).

We also assessed CRCs’ perceptions about their success recruiting prospective participants to clinical research studies based on individual-level factors of prospective participants. The factors were: being from an urban environment vs. rural environment, having worse health status vs. better health status, having English as their primary language vs. English not primary language, being from the same culture and background as the prospective participant vs. from a different culture and background, and having a lower household income vs. higher household income. For each item, CRCs indicated on a scale of 1 to 5 whether they had more success recruiting patients and families from one group (1, e.g., from an urban environment), equal success recruiting people from both groups (3), or more success recruiting patients and families from the other group (5, e.g., from a rural environment).

CRCs also provided demographic information about themselves and information about their role in research. The complete survey instrument is included in the Supplemental Materials.

### Randomized Implicit Bias Vignettes

Implicit bias, by definition, occurs without conscious knowledge. Therefore, it cannot be measured by explicitly asking a respondent about their bias in standard survey questions. Instead, methods such as vignettes or the Implicit Association Test are used to assess implicit bias [29].

We explored implicit bias using vignettes. CRCs read the following two vignettes and were randomly assigned to see either the name Lakisha or Emily for vignette #1 and either José or Joe for vignette #2.

#### Vignette #1

[Lakisha/Emily] is finishing up a clinical visit and may be eligible for a clinical trial that you are recruiting for. They have a family member with them, and they appear to be in a rush to leave. Recruitment for this trial must occur in person during the clinical visit.

#### Vignette #2

[José/Joe] is in the clinic for a clinical visit and based on their chart it looks like they may meet the recruitment criteria for a study you are recruiting for. The study has a lot of follow-up visits and a high-risk profile.

Similar methods have been used to assess implicit bias in hiring practices by randomly assigning the name that appears on resumes [30]. After reading each vignette, respondents indicated how likely they would be to approach the prospective participant (Emily/Lakisha in vignette #1; José/Joe in vignette #2) about recruitment using a 5-point Likert scale. Responses were combined into three categories “Very likely” (Extremely likely and Very likely), “Somewhat likely” (Somewhat likely and Moderately likely), and “Not likely” (Not at all likely).

To examine implicit bias using our vignettes measure, we conducted chi-square analyses to test whether there were differences in CRCs’ likelihood of approaching a prospective participant based on the randomly assigned participant name in the vignette. Using this methodology, evidence of implicit bias would be indicated if CRCs who were randomly assigned to more traditionally White names (Emily and Joe) indicated higher likelihood of approaching the participant for recruitment than CRCs who were randomly assigned to names associated with Black or Hispanic backgrounds (Lakisha and José).

### Survey Administration

CRCs were presented with an information sheet and consent statement, followed by survey items and an implicit bias vignette module administered via Qualtrics. CRCs were permitted to skip any questions they did not wish to answer. CRCs who completed the survey were given the opportunity to enter their email address in a separate form for a chance to be randomly chosen to receive one of four $50 Tango e-gift card incentives. The study was determined to be exempt by the Ann & Robert H. Lurie Children’s Hospital of Chicago Institutional Review Board.

## Results

Surveys were started by 281 people. Among those who started the survey, nine individuals declined to consent, and responses from 52 individuals were removed due to missing data. There were 220 responses analyzed for a survey completion rate of 78% (220/281). Due to the structure of the listservs, a response rate could not be calculated. The majority of respondents were female (67%) and had a bachelor’s degree or less (54%), and more than half of respondents were White (58%) (see Table 1 for full sample demographic characteristics). Respondents worked on a variety of types of clinical research studies, with the most frequent being observational studies (54%) (Table 1). Some respondents reported working on more than one type of research study.

**Table 1.** Sample demographics

Total sample*	*N* = 220
**Gender identity**	% (*n*)
Female	67% (147)
Male	16% (34)
Nonbinary/Other	5% (10)
**Race/Ethnicity**	
Black	4% (8)
Asian	13% (25)
White	58% (107)
Latinx/Hispanic	16% (29)
Other race/eth	4% (7)
Prefer not answer	5% (10)
**Years Experience**	
2-4 years	57% (106)
5+ years	43% (81)
**Education**	
Bachelors or less	54% (103)
Masters or above	46% (88)
**Institution**	
Lurie Children’s Hospital	33% (62)
Northwestern Memorial Health System / Northwestern Univ.	65% (125)
Other	2% (4)
**Research Role**	
Lurie Children’s (*n* = 62)	
Clinical research assistant	5%
Clinical research coordinator I	10%
Clinical research coordinator II	23%
Clinical research coordinator III	23%
Clinical research lead	16%
Other	23%
NMH/NU (*n* = 125)	
Research assistant	18%
Recruitment coordinator	7%
Clinical research coordinator	34%
Project coordinator	15%
Other	27%
Study Type**	
Observational studies	54% (118)
Drug trials	39% (85)
Survey studies	36% (80)
Research registries	35% (77)
Behavioral interventions	26% (57)
Device trials	22% (48)
Other	10% (21)

* Percents may not add to 100% due to rounding.

** Respondents could select more than one study type that they worked on.

### Recruitment Factors

CRCs were asked about five factors that may impact recruitment: language and translations, employee training, participant incentives, study location, and participant research literacy.

### Language and Translations

Nearly a quarter (23%) of CRCs said they always or often have prospective research participants whose preferred language is not English (44% sometimes and 33% rarely/never). However, when approaching families whose preferred language is not English, over half of CRCs said they have translated study documents only sometimes, rarely, or never (60%). Only 40% of respondents said they always or often have study documents that are translated. CRCs reported that having translated study documents resulted in participants being much more willing (27%) or somewhat more willing (37%) to participate in research (29% said no difference, 3% said somewhat less willing, and 4% said much less willing). When asked to rank factors that prevent study documents from being translated, long wait times for internal services (43%) and too few participants requiring translated documents (42%) were the top two factors (see Table 2). When asked about having an interpreter available to help approach families, the majority of CRCs felt that an in-person interpreter was more effective (69%) than a phone interpreter, 27% said they were about the same. Only 4% said a phone interpreter was more effective.

**Table 2.** Top 5 reasons study documents are not translated into participants’ language

	Ranked 1^st^ or 2^nd^ (*n*)	%
Long wait times for internal interpretation	90	43.1%
Too few participants who require translated documents	87	41.6%
Lack of funding	76	36.4%
Study sponsor does not provide translated materials	71	34.0%
The amount of time it takes to submit multiple IRB submissions for approval	68	32.5%

### Employee Training

More than half of CRCs said their institution offers cultural competency training and they have taken this training (60%), 9% said their institution offers training but they have not yet taken it, and 34% said their institution does not offer training. Exploring training by institution, 48% of NU CRCs said they had not yet taken training (47/97), 41% of NMH employees had not (11/27), and 29% of LCH employees had not (18/62). Among CRCs who had completed cultural competency training, 20% said it made them more confident in approaching and recruiting participants who are from a different background, 43% said it did not make a difference, and 36% said it made them less confident.

We conducted follow-up analyses to explore the characteristics of the 34% of CRCs who reported that cultural competency training was not offered (*n* = 72) because it is known that cultural competency training is offered at all institutions involved in the current research. We found that CRCs who did not believe training was offered were primarily White race/ethnicity (51%) and the majority had a bachelor’s degree or higher (56%). They were approximately evenly split in terms of their number of years of experience: 49% had 2–4 years of experience and 51% had 5 or more years of experience. They were involved in a variety of study types (e.g., 54% observational, 42% drug trials, 33% research registries, 31% survey studies, 29% behavioral interventions).

### Participant Incentives

More than half of CRCs indicated that incentives are always or often (44% and 23%, respectively) included in the studies for which they recruit (19% said sometimes, 9% said rarely, and 5% said never). Incentives were generally viewed as having a positive impact on recruitment, with 50% of CRCs reporting that incentives result in potential participants being much more willing to participate in research, 21% said somewhat more willing, 15% said incentives made no difference in willingness to participate, 3% said somewhat less willing, and 1% said much less willing to participate. The top two incentive factors that would improve respondents’ ability to recruit participants were offering gift card incentives for participants’ time and effort (70%) and offering reimbursement for parking and transportation costs (42%) (see Table 3).

**Table 3.** Top 5 factors that would improve recruitment within each domain

	Ranked 1^st^ or 2^nd^ (*n*)	%
**Incentive Factors**		
Offering gift card incentives for participants’ time and effort	140	70.4%
Offering reimbursement for parking and transportation costs	84	42.2%
Offering reimbursement for other study related costs like food and childcare	55	27.6%
Gift cards being available electronically, rather than in physical form	42	21.1%
Participants’ ability to claim gift cards at a wide range of merchant or retailers	42	21.1%
**Location Factors**		
Reduction in the # of required in-person visits	124	67.4%
Option to conduct in-person visits at additional sites/locations	68	37.0%
Option of video visits with physician/staff	47	25.5%
Converting in person data collection to online surveys	46	25.0%
Ability to text participants for scheduling, recruiting, and/or protocol questions	34	18.5%
**Research Literacy Factors**		
Strong relationship between clinician and potential participant	130	71.0%
Good reputation of the research institution	72	39.3%
Education materials related to the study/drug/device	61	33.3%
Videos that explain what research is	58	31.7%
Website or other online resource that explains the study	26	14.2%

### Study Location

Respondents indicated that study location influenced participants’ willingness to participate in research with 28% saying location is always a factor, 48% said often, 19% sometimes, 3% rarely, and 2% said never. The top factors related to study location that respondents indicated would improve their ability to recruit participants were reducing the number of required in-person visits (67%) and including an option to conduct in-person visits at other sites or locations (37%) (see Table 3).

### Research Literacy

A prospective participant’s understanding of research was another factor that respondents indicated impacted recruitment. One quarter of respondents said participants’ understanding of research always impacted their willingness to participate (25%), with 48% saying often, 22% sometimes, and 6% said rarely (no respondents indicated “never”). CRCs indicated that when prospective participants have a greater understanding of research, they are more willing to participate in research, with 49% saying greater understanding makes participants more willing, 45% said somewhat more willing, and 7% said no difference (no CRCs said that greater understanding made participants less willing to participate in research). The top factors related to research literacy that respondents indicated would help them recruit participants were a strong relationship between a clinician and a potential participant (71%) and the research institution having a good reputation (39%) (see Table 3).

### Perceptions of Success Recruiting Participants From Various Groups

**Figure 1.**
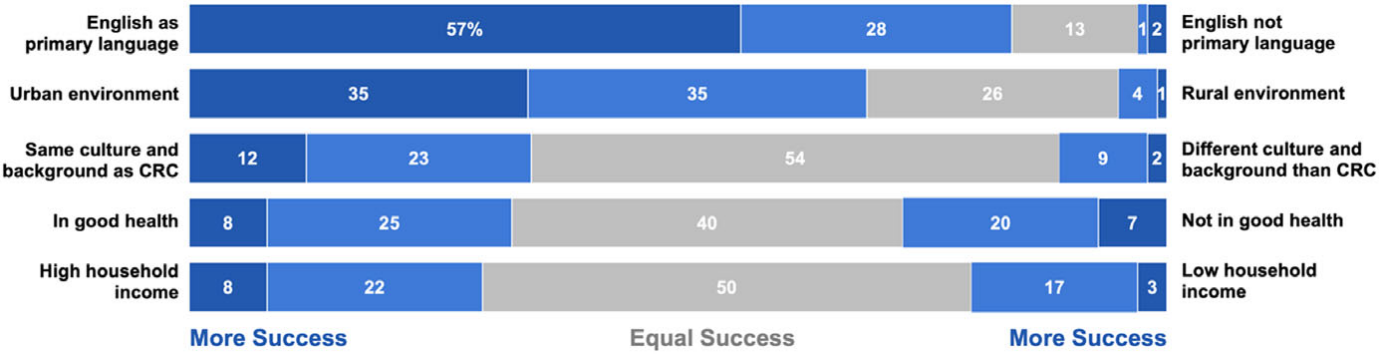
Proportions of respondents who indicated they had more success or equal success recruiting participants from different groups.

CRCs were asked about their success recruiting prospective participants from various groups (Fig. 1). In general, CRCs indicated that they were more successful recruiting participants with higher household income (vs. lower household income), who were from the same culture and background as themselves (vs. a different culture and background), who spoke English as a primary language (vs. English non-primary language), and who were from urban environments (vs. rural environments). Success recruiting participants who were in better health was similar to success recruiting those who were in worse health. Taken together, these findings indicate that CRCs perceived themselves to be more successful recruiting prospective participants who are from groups that are not underrepresented in research.

We explored whether differences emerged in perceptions of success recruiting participants from different groups based on characteristics of the CRCs such as the CRC’s race/ethnicity, education level, gender, years of experience, and whether there was an approved treatment for the condition under study using chi-square analyses. The only analysis that indicated a significant difference showed that CRCs who had more experience in clinical research (5+ years) were more likely to say they had greater success recruiting participants from their same culture and background (43%) compared with CRCs who had less experience (2–4 years) (27%), *p* < .05.

Two additional analyses emerged as marginally significant (*p* < .10). First, non-White CRCs were twice as likely to say they had greater success recruiting participants who did not have English as their primary language (16%) compared with White CRCs (8%) (note that due to small sample subsample sizes, we combined respondent race/ethnicity into White and non-White for this analysis). Second, when a study medical condition did not have an approved treatment, CRCs were more likely to say they had more success recruiting participants from their CRC’s same culture or background (49%) than if there was an approved treatment (32%). There were no differences in perceptions of success in recruitment based on the CRC’s institution (LCH or NU/NMH). We did not use a correction for multiple comparisons for these analyses.

### Implicit Bias

**Figure 2.**
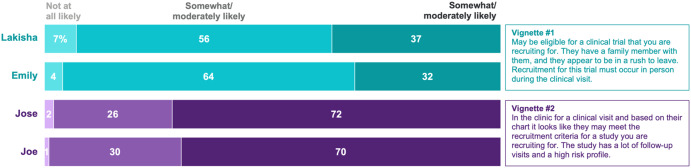
Likelihood of approaching the patient for recruitment by patient name. Percentages may not add to 100% due to rounding.

Chi-square analyses did not indicate a significant difference in the likelihood of approaching for recruitment based on the name in the vignette. For instance, the proportion of CRCs who were very or extremely likely to approach Lakisha for recruitment (37%) did not differ significantly from the proportion who were very or extremely likely to approach Emily (32%) (*p* = .41). Similarly, the proportion of CRCs who were very or extremely likely to approach José for recruitment (72%) did not differ significantly from the proportion who were very or extremely likely to approach Joe (70%) (*p* = .72) (Fig. 2). We also explored whether years of experience was associated with intentions to approach for recruitment and found that whether a CRC had more (5+ years) or less (2-4 years) clinical research experience was not associated with differences in intentions to recruit by vignette name for either the Lakisha/Emily vignette or the José/Joe vignette (all *ps* > .05).

## Discussion

This study explored CRCs perspectives about barriers and facilitators to research recruitment. One of the important themes that emerged was the impact of participants’ language preferences on recruitment. The majority of CRCs reported that having translated study materials improved recruitment among prospective participants whose preferred language is not English, but study documents are not regularly translated. CRCs also indicated that having an in-person interpreter was more effective than using a phone interpreter for recruitment. Our findings are consistent with other research that has indicated language is a frequently cited barrier to recruitment [19,22,31]. A review of pediatric studies found that Latino and Asian/Pacific Islander caregivers were more likely to participate if the study materials were in their preferred language [32].

Incentives and study logistics also were of critical importance for recruitment. CRCs generally felt that incentives boosted recruitment. Being able to offer gift cards to compensate participants for their time and reimburse transportation costs were the top incentive factors to improve recruitment. This is in line with previous research that has suggested that financial incentives can be used to boost recruitment among underrepresented groups, specifically Hispanic participants [33]. A recent thematic review also noted that increased benefits such as reimbursements for transportation costs and monetary incentives are a potential mechanism to increase representation of people from racial and ethnic minority groups [21]. CRCs also reported that study location frequently factored into participants’ willingness to participate in clinical research studies. CRCs suggested that reducing the number of in-person visits would improve recruitment. This aligns with perspectives of prospective participants in other studies, citing logistics, time, and transportation as a barrier to research participation [19,31,34].

Consistent with other work on this topic, research literacy was connected to recruitment success [35]. CRCs indicated that when prospective participants have greater understanding of research, they are more willing to participate. Importantly, a top factor to improve recruitment was a strong relationship between the clinical provider and the prospective participant. The relationship between provider and participant has not been a primary topic of previous research on facilitators of clinical research study recruitment. Future research would benefit from further exploring ways to promote positive, trusting relationships between physician scientists and prospective participants.

CRCs reported having greater success in their own recruitment endeavors when prospective participants were from groups that are not underrepresented in research (e.g., English speakers and urban dwellers). However, our measure of implicit bias did not indicate a difference in likelihood of approaching a prospective participant depending on whether the name of the participant was more traditionally White or less traditionally White. These two findings taken together are somewhat paradoxical because they indicate that CRCs have explicit awareness of differential success in their actual recruitment endeavors based on characteristics of prospective participants, but CRCs did not show an implicit bias for recruiting dominant groups more than underrepresented groups in our vignette measure. It is possible that our vignette measure did not adequately capture CRCs’ implicit bias. Previous research has found that bias and stereotyping of minority participants can occur among research teams, including research staff, physicians, and principal investigators [25]. There is also extensive literature documenting the impact of bias in clinical decision making and how it may contribute to care and outcome disparities; however, a 2017 systematic review found that only two of nine studies found an association between physicians’ implicit bias (IAT score) and clinical decision making (e.g., in clinical vignettes) [24,36,37]. It would be beneficial for future research to explore a wider range of vignettes with different situational framing such as the study team being busy or enrollment being close to completion. The characteristics of the potential participant (e.g., race/ethnicity) in the vignettes would be randomly assigned. This would help researchers determine whether there is utility in vignette measures of implicit bias in clinical trial recruitment.

Our findings related to cultural competency training indicate a need for additional research on effective training strategies for research staff. Cultural competency training was freely available at both institutions. Most CRCs in the present study had completed some form of cultural competency training at their institution. However, among those who completed training, more than half felt that the training either had no effect or had a counter-productive effect, making them feel less confident in approaching prospective participants from backgrounds different than their own. There is evidence that research teams believe cultural competency training may be helpful for recruitment of underrepresented groups, but few studies have evaluated the impact of this type of education on actual enrollment [38]. One study found that the minority recruitment rate at sites where staff completed cultural competency training was not significantly different from recruitment rates at sites that did not complete the training [39]. More research is needed to determine whether cultural competency training is associated with improved recruitment of underrepresented populations, and what factors are associated with more successful training programs.

The current study is not without limitations. Specifically, the proportion of CRCs who were non-White was relatively low, limiting our ability to conduct subgroups analyses to explore differences in perceptions among groups. We also were not able to calculate a response rate due to the structure of the clinical research listservs used in recruitment for this study. Additionally, our data do not permit us to link responses on the current survey to actual recruitment numbers to explore, for example, how the perceptions of CRCs map on to their actual recruitment approach behaviors and successes and challenges in recruitment. With respect to cultural competency training, we do not know specific details of the trainings that CRCs completed and therefore we cannot know what elements of training are more or less helpful for improving recruitment of underrepresented groups. Finally, the current study focused primarily on underrepresented groups based on race/ethnicity and primary language, but there are other groups that may be underrepresented in research such as women, children and youth, older adults, and LGTBQ + individuals. Future research should evaluate the extent to which other groups are underrepresented in clinical research, and identify barriers and facilitators to their research participation.

## Supporting information

Heffernan et al. supplementary material Heffernan et al. supplementary material

## Data Availability

Deidentified individual participant data will not be made available.

## Supplementary material

The supplementary material for this article can be found at https://doi.org/10.1017/cts.2023.611.
