# Supplementary material for: Barriers and facilitators to recruitment of underrepresented research participants: Perspectives of clinical research coordinators
Source: J Clin Transl Sci. 2023 Aug 18;7(1):e193. doi: 10.1017/cts.2023.611 (PMC10514687; doi:10.1017/cts.2023.611)
Supplement: Heffernan et al. supplementary material [file S2059866123006118sup001.docx]

[PrefLang_Freq]
**First, we’d like to ask you a bit about factors that can influence approaching and recruiting research participants who are traditionally underrepresented in research.**
   How often do you have prospective research participants whose preferred language is not English?

- Always
- Often
- Sometimes
- Rarely
- Never

[Translation_Freq] When approaching families whose primary language is not English, how often are the participant facing study documents translated into their preferred language?

- Always
- Often
- Sometimes
- Rarely
- Never

[Translation_Impact] When approaching a participant whose preferred language is not English, does having translated study documents impact their willingness to participate in research studies?

- Much more willing to participate
- Somewhat more willing to participate
- No difference
- Somewhat less willing to participate
- Much less willing to participate

[Translation_Factor] Please rank the factors that prevent study documents from being translated into a participant’s preferred language. Please rank the factors, with 1 being most impactful.

______ Lack of funding

______ Long wait times from internal services

______ Too few participants who require translated documents

______ The amount of time it takes to submit multiple IRB submissions for approval

______ Study sponsor does not provide translated materials

______ Other:

[Interpreter_Method] When using an interpreter to approach patients and families, is an in-person interpreter or a phone interpreter more effective in communicating?

- In person interpreter is more effective
- In person and phone interpreter are about the same
- Phone interpreter is more effective

[PrefLang_Other] Is there anything else you'd like to tell us about the barriers or facilitating factors that impact your ability to approach and recruit participants whose preferred language is not English?

________________________________________________________________

[Training] Does your institution provide cultural competence training?

- Yes, and I completed this training
- Yes, but I haven't completed this training
- No

[Training_Impact] Did the cultural competence training impact your confidence in approaching and recruiting participants who are from a different culture or background than yourself?

- Much more confident
- Somewhat more confident
- No difference
- Somewhat less confident
- Much less confident

[Incentive_Freq] When approaching families about a study, how often does the study include incentives or reimbursement for participant’s time and effort?

- Always
- Often
- Sometimes
- Rarely
- Never

[Incentive_Impact] Does having study incentives or reimbursement for participant’s time and effort impact their willingness to participate?

- Much more willing to participate
- Somewhat more willing to participate
- No difference
- Somewhat less willing to participate
- Much less willing to participate

[Incentive_Factor] Please rank the factors related to incentives and reimbursement that would improve your ability to recruit participants. Please rank the factors, with 1 being most impactful.

______ Offering gift card incentives for participant's time and effort

______ Gift cards being available electronically, rather than in physical form

______ Participant’s ability to claim gift cards at a wide range of merchants or retailers

______ Offering tiered incentives based on the participant’s needs

______ Offering reimbursement for parking and transportation costs

______ Offering reimbursement for other study related costs like food and childcare

______ Other:

[Location_Freq] When approaching families about a study, how often does the location of the study visits influence their willingness to participate?

- Always
- Often
- Sometimes
- Rarely
- Never

[Location_Factor] Please rank the factors related to location of study visits that would improve your ability to recruit participants. Please rank the factors, with 1 being most impactful.

______ Reduction in the number of required in-person visits

______ Option to conduct in-person visits at additional sites/locations

______ Options for remote monitoring (wearable sensors, remote vital sign collection)

______ Devices to remotely measure adherence or compliance

______ Option for video visits with physician/staff

______ Ability to text participants for scheduling, recruiting, and/or protocol questions

______ Home collection of biospecimens

______ Converting in –person data collection to online surveys

______ Utilizing videography and digital photography to collect information

______ Other:

[Understanding_Freq] When approaching families about a study, how often does a potential participant’s understanding of research impact their willingness to participate?

- Always
- Often
- Sometimes
- Rarely
- Never

Q20 Does a participant having a strong understanding of research impact their willingness to participate?

- Much more willing to participate
- Somewhat more willing to participate
- No difference
- Somewhat less willing to participate
- Much less willing to participate

Q21 Please rank the factors related to understanding of research that would improve your ability to recruit participants into the box on the right. Please rank the factors, with 1 being most impactful.

______ Videos that explain what research is

______ Educational materials related to the study/drug/device

______ Strong relationship between clinician and potential participant

______ Good reputation of the research institution

______ Advertising by the sponsor or study team

______ Website or other online resource that explains the study

______ Other: ______

[RG_LSS_Other] Is there anything else you'd like to tell us about the barriers or facilitating factors that impact your ability to approach and recruit participants who are traditionally underrepresented in research (minority race/ethnicity, low socioeconomic status, non-English speaking, rural geography)?

________________________________________________________________

End of Block: I. Facilitators & Barriers

Start of Block: II. Brief Implicit Bias Measure

[Exp_RG]
**Next, we’d like to know about your experience recruiting potential participants at your organization.**
 **For each of the items below, please indicate which group you have had more success recruiting by choosing a point on the scale. There is no right or wrong answer. The middle point indicates equal success for both groups.**

|  |  |  | Equal success for both groups |  |  |  |
| --- | --- | --- | --- | --- | --- | --- |
|  |  |  |  |  |  |  |
| More success recruiting patients and families who are from urban environments |  |  |  |  |  | More success recruiting patients and families who are from rural environments |

[Exp_HS]

|  |  |  | Equal success for both groups |  |  |  |
| --- | --- | --- | --- | --- | --- | --- |
|  |  |  |  |  |  |  |
| More success recruiting patients who have worse health status |  |  |  |  |  | More success recruiting patients who have better health status |

[Exp_NES]

|  |  |  | Equal success for both groups |  |  |  |
| --- | --- | --- | --- | --- | --- | --- |
|  |  |  |  |  |  |  |
| More success recruiting patients and families for whom English is the primary language |  |  |  |  |  | More success recruiting patients and families for whom English is not the primary language |

[Exp_RE]

|  |  |  | Equal success for both groups |  |  |  |
| --- | --- | --- | --- | --- | --- | --- |
|  |  |  |  |  |  |  |
| More success recruiting patients and families from the same culture and background as myself |  |  |  |  |  | More success recruiting patients and families from a different culture and background than myself |

[Exp_LSS]

|  |  |  |  |  |  |  |
| --- | --- | --- | --- | --- | --- | --- |
|  |  |  |  |  |  |  |
| More success recruiting patients and families who have lower household income |  |  |  |  |  | More success recruiting patients and families who have higher household income |

End of Block: II. Brief Implicit Bias Measure

Start of Block: III. Brief Implicit Bias Measure

[Vignette_Intro] **In this next section, we are looking at how certain study and clinical circumstances may or may not be connected with recruitment. You’ll read a brief paragraph about a potential recruitment scenario and answer a question after each.**

**Note** Respondents randomly assigned to receive *either* “Emily” or “Lakisha” vignette.

End of Block: III. Brief Implicit Bias Measure

Start of Block: Vignette 1EM patient/study level factor (in a hurry, recruitment in person only)

V1EM Emily is finishing up a clinical visit and may be eligible for a clinical trial that you are recruiting for. They have a family member with them, and they appear to be in a rush to leave. Recruitment for this trial must occur in person during the clinical visit.

How likely would you be to approach Emily about recruitment?

- Not at all likely
- Somewhat likely
- Moderately likely
- Very likely
- Extremely likely

End of Block: Vignette 1EM patient/study level factor (in a hurry, recruitment in person only)

Start of Block: Vignette 1LA patient/study level factor (in a hurry, recruitment in person only)

V1LA Lakisha is finishing up a clinical visit and may be eligible for a clinical trial that you are recruiting for. They have a family member with them, and they appear to be in a rush to leave. Recruitment for this trial must occur in person during the clinical visit.

How likely would you be to approach Lakisha about recruitment?

- Not at all likely
- Somewhat likely
- Moderately likely
- Very likely
- Extremely likely

End of Block: Vignette 1LA patient/study level factor (in a hurry, recruitment in person only)

Start of Block: Vignette 2 Jose: study/clinical level factor (extensive follow-up, risk profile)

**Note** Respondents randomly assigned to receive *either* “José” or “Joe” vignette.

V2Jose José is in the clinic for a clinical visit and based on their chart it looks like they may meet the recruitment criteria for a study you are recruiting for. The study has a lot of follow-up visits and a high risk profile.

How likely would you be to approach José about recruitment?

- Not at all likely
- Somewhat likely
- Moderately likely
- Very likely
- Extremely likely

End of Block: Vignette 2 Jose: study/clinical level factor (extensive follow-up, risk profile)

Start of Block: Vignette 2 Joe: study/clinical level factor (extensive follow-up, risk profile)

V2Joe Joe is in the clinic for a clinical visit and based on their chart it looks like they may meet the recruitment criteria for a study you are recruiting for. The study has a lot of follow-up visits and a high risk profile.

How likely would you be to approach Joe about recruitment?

- Not at all likely
- Somewhat likely
- Moderately likely
- Very likely
- Extremely likely

End of Block: Vignette 2 Joe: study/clinical level factor (extensive follow-up, risk profile)

Start of Block: III. Demographic Section

[Dem_Intro]
**Finally, we’d like to ask you a few questions about yourself.**

[Gender]
To which gender identity do you most identify?

- Female
- Male
- Transgender
- Non-binary/non-conforming
- Other: __________________________________________________
- Prefer not to respond

[Race_Ethnicity] What race(s) or ethnicity do you consider yourself to be? Please select all that apply.

- American Indian or Alaska Native
- Asian
- Black or African American
- Hispanic, Latinx, or Spanish
- Middle Eastern or North African
- Native Hawaiian or Other Pacific Islander
- White
- Some other race or ethnicity __________________________________________________
- I prefer not to answer

[Other_Language] Are you fluent in any languages, other than English?

- Yes
- No

[Language_Specify] What language(s) are you fluent in?

- Arabic
- Chinese Cantonese
- Chinese Mandarin
- German
- Greek
- Gujarati
- Hindi
- Italian
- Korean
- Polish
- Russian
- Spanish
- Tagalog
- Urdu
- Vietnamese
- Other: __________________________________________________

[Education] What is the highest degree or level of education that you’ve completed?

- High School Diploma
- Associates Degree
- Bachelor’s Degree
- Registered Nurse (RN)
- Licensed Practical Nurse (LPN)
- Master’s Degree
- Advance Practice Nurse (APN) or Nurse Practitioner (NP)
- Medical Doctor (MD)
- Doctor of Osteopathic Medicine (DO)
- Doctor of Philosophy (PhD)
- Other: __________________________________________________

[Certificate] Do you have a research certificate, such as an ACRP or SOCRA certification?

- Yes
- No

[Institution] At which institution do you currently work?

- Lurie Children’s Hospital
- Northwestern Memorial Hospital (downtown Chicago)
- Northwestern Medicine Central DuPage
- Northwestern Medicine Lake Forest Hospital
- Northwestern University
- Shirley Ryan Ability Lab
- Other: __________________________________________________

NU_Role What is your role? *Please specify*

- Research Assistant
- Program Assistant
- Clinical Research Coordinator
- Project Coordinator
- Research Study Recruitment Coordinator
- Regulatory Coordinator
- Other __________________________________________________

LCH_Role What is your role?

- Clinical Research Assistant
- Clinical Research Coordinator I
- Clinical Research Coordinator II
- Clinical Research Coordinator III
- Clinical Research Lead
- Research Scientist
- Other __________________________________________________

[Trial_Type] What type of studies do you currently support? Please select all that apply.

- Drug trials
- Device trials
- Observational studies (no treatment or intervention)
- Behavioral interventions
- Research registries
- Survey studies
- Other: __________________________________________________

[Responsibilities] What study tasks are you typically responsible for? Please select all that apply.

- Study design/protocol writing
- Regulatory submissions/study start-up
- Identifying eligible participants
- Recruiting, consenting, and enrolling participants
- Retention and engagement of participants
- Data collection
- Data analysis
- Other: __________________________________________________

[Funding_Source] What are the main funding sources for studies that you support? Please select all that apply.

- Pharmaceutical
- Philanthropic/Foundations
- Federal
- Non-Federal/Other Organization
- Internal/Institutional
- Other: __________________________________________________

[Experience_Years] How many years have you been working in clinical research?

- 2-4 years
- 5-7 years
- 8-10 years
- 11 or more years

[Participant_Age] What is the age range for the participants that you recruit and/or study?

_______ Youngest

_______ Oldest

[Condition] Do you recruit and/or study research participants with a specific condition(s)?

- Yes
- No

[Condition_Specify] What condition(s) do the research participants have? Please list all that apply.

________________________________________________________________

[Approved_Treatment] Is there an effective/approved treatment for this condition(s)?

- Yes
- No

[Recruit_Healthy] Do you recruit healthy volunteers for your studies?

- Yes
- No

End of Block: III. Demographic Section

Start of Block: Gift Card

GIft card Are you interested in entering a raffle for a $50 gift card? By clicking, “Yes” below you will be directed to a separate survey to enter your email address. As a reminder, that information will not be linked to your survey responses.

- Yes, I am interested in entering the raffle
- No, I am not interested in entering the raffle
